# Essential Role of NK Cells in IgG Therapy for Experimental Autoimmune Encephalomyelitis

**DOI:** 10.1371/journal.pone.0060862

**Published:** 2013-04-05

**Authors:** Wai Po Chong, Man To Ling, Yinping Liu, Rachel R. Caspi, Wai Man Wong, Wutian Wu, Wenwei Tu, Yu Lung Lau

**Affiliations:** 1 Department of Paediatrics and Adolescent Medicine, Li Ka Shing Faculty of Medicine, The University of Hong Kong, Pokfulam, Hong Kong SAR, China; 2 Laboratory of Immunology, National Eye Institute, Bethesda, Maryland, United States of America; 3 Department of Anatomy, Li Ka Shing Faculty of Medicine, The University of Hong Kong, Pokfulam, Hong Kong SAR, China; University Hospital of Heidelberg, Germany

## Abstract

Intravenous immunoglobulin has long been used in treating autoimmune diseases, although mechanisms remain uncertain. Activating Fcγ receptors are receptors of IgG and reported to be essential in intravenous immunoglobulin (IVIG) therapy. Therefore, we hypothesized natural killer (NK) cells, which express abundant activating Fcγ receptors, are the potential cellular target. In experimental autoimmune encephalomyelitis (EAE), we demonstrated that IgG suppressed disease development in intact, but not in NK cell depleted mice. Adoptive transfer of IgG-treated NK cell could protect mice against EAE, and suppressed interferon γ and interleukin 17 production. The percentage of CD4^+^Foxp3^+^ regulatory T cells was significantly increased. The increase of regulatory T cells was also observed in IgG-treated EAE mice but not in NK cell depleted mice. In vitro experiments confirmed that IgG-treated NK cells enhanced regulatory T cell induction from naïve CD4^+^ T cells. Interestingly, cells from draining lymph nodes produced more interleukin 2 after the adoptive transfer of IgG-treated NK cells. We neutralized interleukin 2 and the induction of CD4^+^Foxp3^+^ T cells by IgG-treated NK cells was significantly reduced. To our knowledge, we identified for the first time the critical role of NK cells in the mechanism of IgG-induced induction of Treg cells in treatment of autoimmunity.

## Background

Intravenous immunoglobulin (IVIG) is IgG purified from pooled blood plasma of healthy donors. Its administration was originally designed as replacement therapy for antibody deficiencies [1]. Since then, high dose IVIG has been established as an important treatment of autoimmune diseases including multiple sclerosis, chronic inflammatory demyelinating polyneuropathy, Guillain-Barr'e syndrome and myasthenia gravis [1]. The protective effects of IVIG were also reported in animal studies including experimental autoimmune encephalomyelitis (EAE) [2], arthritis [3] and type I diabetes [4]. Although the use and beneficial effects of IVIG in autoimmune diseases are well documented, the mechanisms remain unclear. Fcγ receptors were suggested as the potential target for IVIG treatment, as they are the receptors of IgG [1]. Siragam et al. confirmed the critical role of activating Fcγ receptors in the anti-inflammatory effects of IVIG *in vivo* [5]. In an invariant NKT(iNKT) cell-mediated allergic airway inflammation model, IVIG regulates iNKT cells through activating Fcγ receptor, FcγRIIIa [6]. A recent study also confirmed that the inhibitory effect of IVIG on T cells responses is independent of the inhibitory receptor FcγRIIb, supporting the role of activating Fcγ receptors in IVIG therapy [7]. Therefore, cells expressing activating Fcγ receptor could be the cellular targets for IVIG treatment.

Natural killer (NK) cells were previously thought as the effectors in innate immunity in direct killing of transformed and virus infected cells. They are responsible for triggering inflammation by releasing pro-inflammatory cytokines and chemokines rapidly. However, more recent studies found that they can reciprocally interact with dendritic cells (DCs) [8] and regulate adaptive immunity by modulating T cell proliferation and polarization [9], [10]. More recent data also suggest that NK cells are capable of interacting with both regulatory T (Treg) cells and B cells [11], [12]. As NK cells express high level of activating Fcγ receptors that could recognize IgG [13], we hypothesized that NK cells are the cellular targets for IVIG treatment in regulating T cell immune responses and hence, T cell mediated autoimmune diseases. Using EAE as an *in vivo* T cell-mediated autoimmune animal model, we found that high dose of human IgG treatment protected mice from EAE but was ineffective in NK cell depleted mice. Conversely, adoptive transfer of IgG-treated NK (IgG-NK) cells could suppress EAE through induction of CD4^+^Foxp3^+^ Treg cells. Our *in vitro* experiments further demonstrated that IgG-treated NK cells induced CD4^+^Foxp3^+^ Treg cells in the presence of interleukin (IL)-2 and transforming growth factor (TGF)-β1, providing a mechanistic basis for this phenomenon.

## Results

### IgG protects mice from EAE and suppresses their IL-17 and IFN-γ production in normal, but not in NK cell-depleted mice

**Figure 1:**
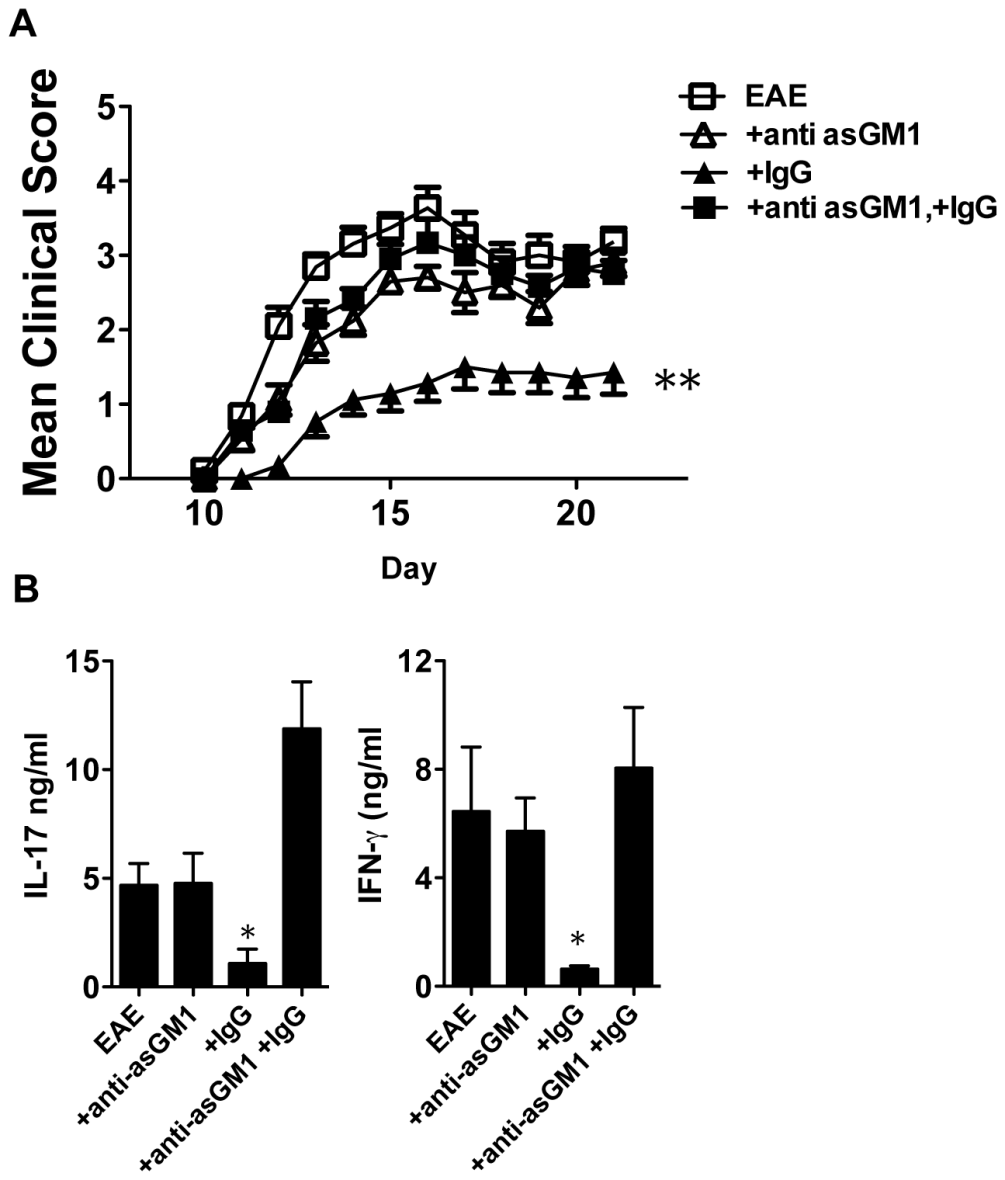
IgG protects NK sufficient, but not NK depleted mice from EAE and lowers associated immunological responses. EAE was induced in C57BL/6N mice as described in Materials and Methods. IgG was injected intravenously at day 0 and 4. NK cell was depleted in EAE mice with or without IgG treatment by injecting anti-asialo GM1 antibody intravenously 1 day before and 3 days after the immunization. (**A**) **EAE development**. NK depleted or non-depleted mice were followed for EAE and disease was scored as described in Methods (n = 16–20). (**B**) **Cytokine production.** On day 10, cells were isolated from draining lymph nodes. 2.5×10^6^ isolated cells were re-stimulated with MOG_35–55_ (20 µg/ml) for 72 hours. Supernatant was collected and cytokine production was determined (n = 4–7). Data are pooled from 4 independent experiments (**A**) and 2 independent experiments (**B**) and displayed as mean ± SEM. *: p<0.05; **: p<0.01, Krusaki-Wallis test.

To test our hypothesis that NK cells are the cellular targets for IVIG treatment, we first determined whether NK cells are required for efficacy of IgG treatment against EAE. Treatment with anti-asialo GM1 antibody depleted more than 90% of NK cells in different tissues, i.e. blood, spleen and lymph nodes, as confirmed by FACS (Figure S1). We then applied 2 doses of high dose of human IgG to EAE mice with or without NK cell depletion by anti-asialo GM1 antibody on day 0 and 4 relative to immunization. As reported previously, 2 doses of IgG treatments could significantly suppress EAE development [14]. IgG could significantly suppress EAE in our experiments, confirming these data. Importantly, EAE protection was not observed in NK cell depleted mice (p<0.01, Figure 1A). To demonstrate the protective effect is specific for IgG, we compared EAE development in IgG treated group to a control group that is treated with another serum protein, i.e. BSA. We observed that BSA treated EAE mice also developed severe EAE (Figure S2) but not in IgG treated mice.

It has been reported that IVIG could suppress the production of two known pathogenic cytokines, IL-17 and interferon (IFN)-γ, in EAE mice [15]. At day 10, we isolated the cells from draining lymph nodes of EAE mice with or without NK cell depletion after IgG treatment and studied their MOG_35–55_ specific IL-17 and IFN-γ production. We found that both these pathogenic cytokines were suppressed after IgG treatment but again, this was not observed in NK cell-depleted mice (p<0.05, Figure 1B). Collectively, our data are consistent with observations of previous studies [2], [14], [15] that IgG could suppress EAE as well as the production of pathogenic cytokines. Importantly, we demonstrated that this suppression requires the presence of NK cells.

### Adoptive transfer of IgG-NK cells suppresses disease development, as well as IL-17 and IFN-γ production in EAE

**Figure 2:**
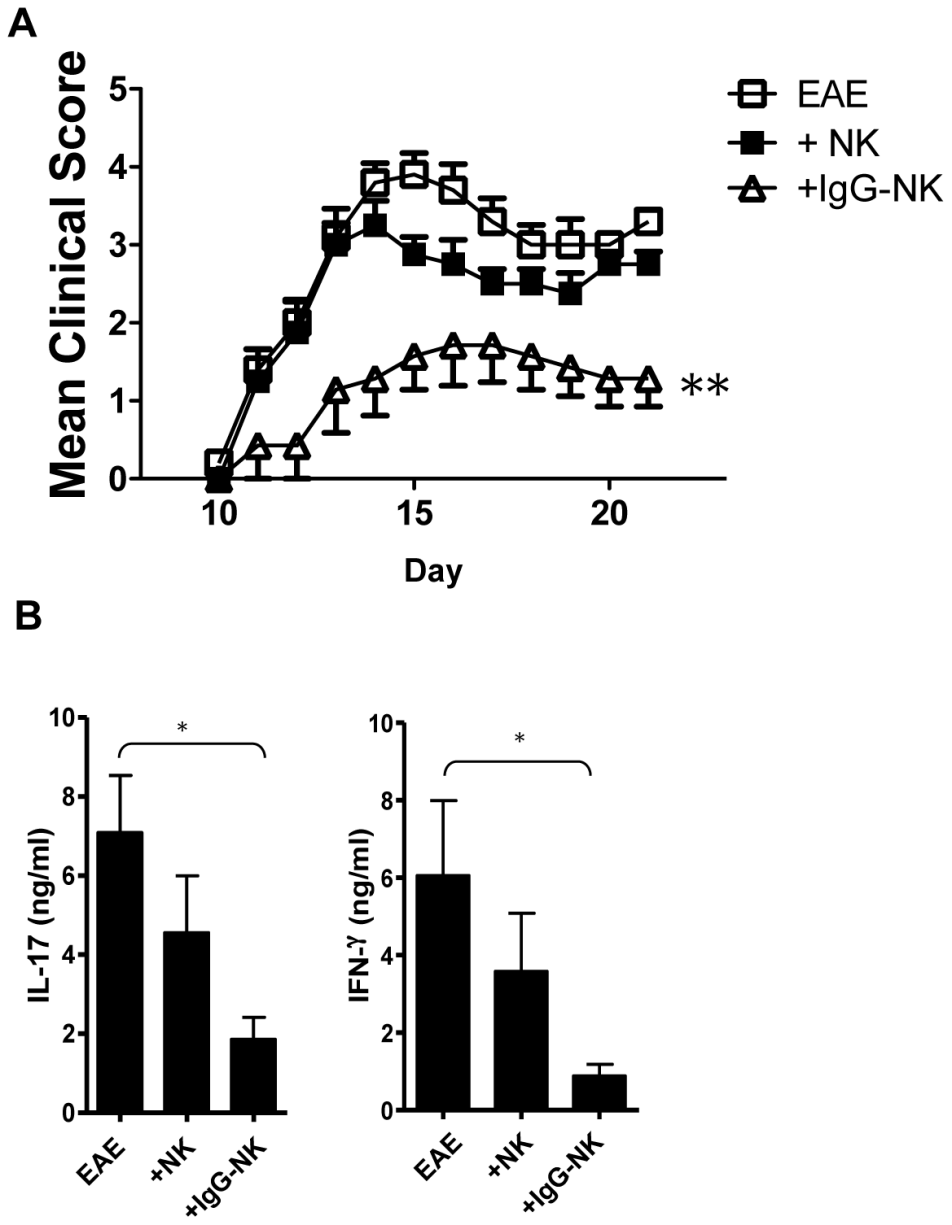
Adoptive transfer of IgG-NK cell suppresses EAE. EAE was induced in C57BL/6N mice. NK cells were isolated from the spleen of naïve mice and incubated with IgG for 18 hours. 1×10^6^ of IgG-NK cells or untreated-NK cells were injected intravenously on the day of EAE induction. (**A**) The mean clinical scores were significantly decreased after injection of IgG-NK cell when compared with untreated EAE mice (p<0.01). No significant difference was observed between untreated EAE mice and EAE mice with adoptive transfer of untreated-NK cells (n = 10–11). (**B**) At day 10, cells were isolated from draining LNs. 2.5×10^6^ isolated cells were re-stimulated with MOG_35–55_ for 72 hours. Cytokine productions were determined (n = 4–7). Data are pooled from 3 independent experiments (**A**) and 2 independent experiments (**B**) and displayed as mean ± SEM. *: p<0.05; **: p<0.01, Krusaki-Wallis test.

We next hypothesized that IgG-NK cells alone would also suppress EAE. We isolated NK cells from the spleen of naïve C57BL/6N mice and pre-treated them with IgG and adoptively transferred 1×10^6^ IgG-NK cells or untreated NK cells to mice at the day of EAE immunization. Interestingly, we observed that IgG-NK cells protected mice from EAE similarly to IgG treatment (p<0.01, Figure 2A) but untreated NK cells did not.

Both Th17 and Th1 effector responses are involved in EAE development [16]–[18]. Therefore, we examined the antigen specific cytokine productions of IL-17 and IFN-γ to MOG_35–55_ of EAE mice after adoptive transfer of IgG-NK cells. We isolated cells from the draining lymph nodes and stimulated them with MOG_35–55_. Both IL-17 and IFN-γ production was significantly reduced after IVIG- NK cell infusion (p<0.05, Figure 2B) but no significant reduction was observed after adoptive transfer of untreated NK cells (p>0.05, Figure 2B). For production of other cytokines that are involved in inflammation and T cell polarization, i.e. IL-4, IL-6, IL-10, tumor necrosis factor (TNF)-α and transforming growth factor (TGF)-β1, no statistically significant difference was observed (Figure S3) between responses of IgG-NK *vs* NK infused mice.

### IgG and IgG-NK cells treatments reduce demyelination in EAE mice

Toluidine blue staining sections were used to investigate the demyelination of EAE mice with different treatments (Figure S4). Demyelination was apparent in untreated EAE mice (Figure S4A) but not in IgG-treated EAE mice (Figure S4B). However, demyelination was present in NK cell depleted mice despite IgG treatment (Figure S4D). This further confirms the importance of NK cells in IgG treatment *in vivo*. Conversely, in EAE mice that received adoptive transfer of IgG-NK cells (Figure S4F), more myelinated axons were found when compared with control EAE mice. These observations are in line with data in Figure 1 and 2 that clinical manifestations of EAE were suppressed in mice after these two treatments.

### IgG induces CD4^+^Foxp3^+^ Treg cells in EAE mice and it requires NK cells

**Figure 3:**
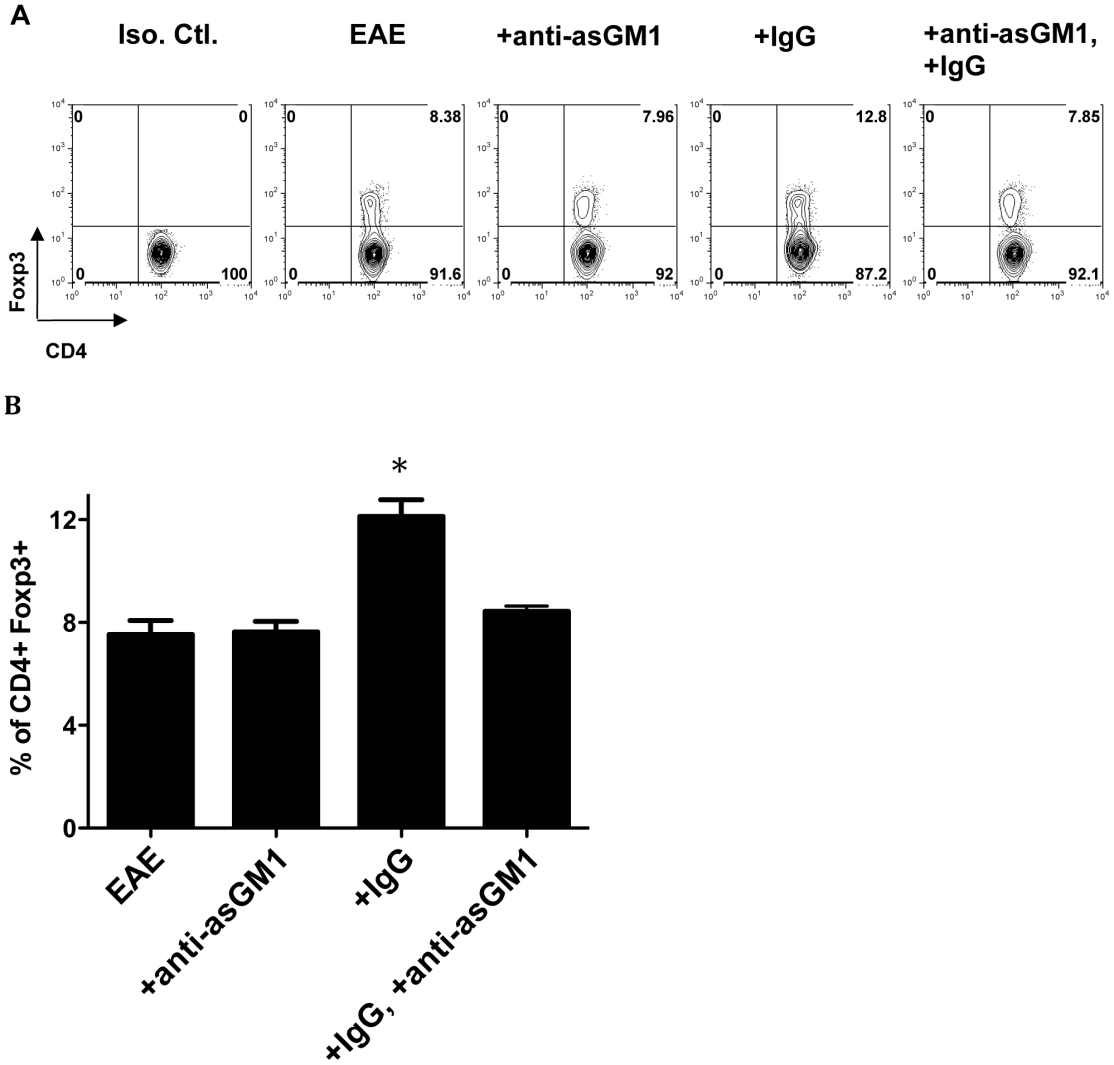
IgG could not induce CD4^+^Foxp3^+^ Treg cells in NK cell-depleted EAE mice. (**A, B**) EAE was induced in C57BL/6N mice. IgG was injected intravenously at day 0 and 4. NK cell was depleted in EAE mice with or without IgG treatment by injecting anti-asialo GM1 antibody intravenously 1 day before and 3 day after the immunization. Cells were isolated from draining lymph nodes of EAE mice 10 days after EAE induction. Anti-CD4 antibody was used to stain the surface expression of CD4. Foxp3 expression on gated CD4 cells was determined by intracellular staining. Mice: n = 6 per group. Data are representative of at least 2 independent experiments and displayed as the mean ± SEM. Krusaki-Wallis test was used for comparison. *: p<0.05.

A previous study reported that IgG suppresses EAE by inducing Treg cells [15]. We confirmed that IgG could induce CD4^+^Foxp3^+^ Treg cells in EAE mice (p< 0.05, Figure 3). However, this increase was not observed in EAE mice after NK cell depletion (p<0.05, Figure 3), which supports that IgG therapy requires the presence of NK cells and suggests that IgG acts through NK cells to induce Tregs.

**Figure 4:**
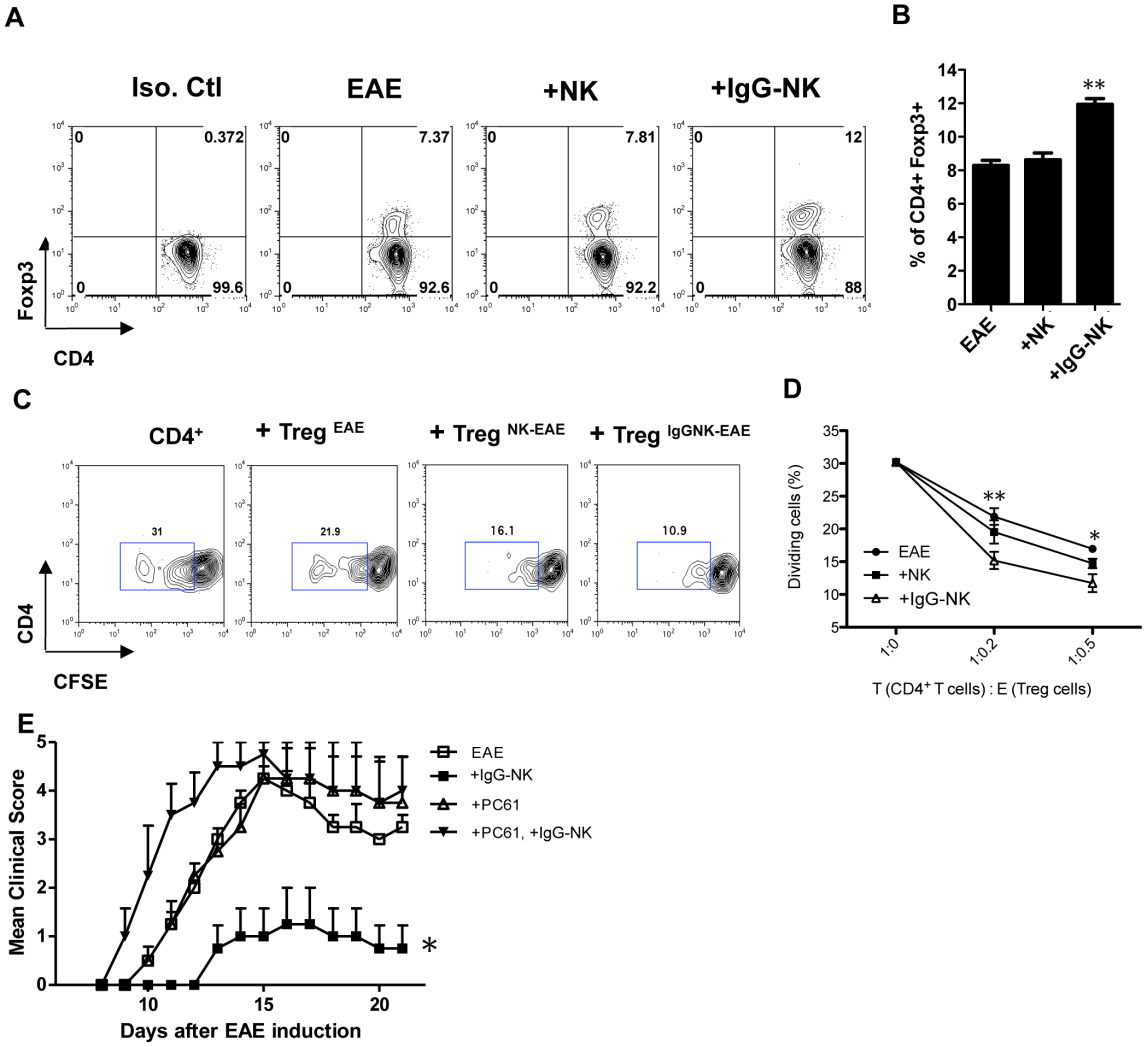
IgG-NK cells suppress EAE by inducing CD4^+^Foxp3^+^ Treg cells with stronger inhibitory effect. EAE was induced in C57BL/6N mice and 1×10^6^ of IgG-NK cells or untreated-NK cells were injected intravenously at the day of EAE induction. (**A, B**) Cells were isolated from draining LNs 10 days after EAE induction and analyzed by Flow cytometry. Treg cells were identified by intracellular expression of Foxp3 on the gated CD4^+^ cells. Data are displayed as the mean percentage ± SEM of the combined data with 4–6 mice per group from 2 independent experiments. (**C, D**) 2×10^4^ CFSE-labeled CD4^+^CD25^−^ T cells from the spleen of immunized EAE mice were cocultured with 1×10^4^ irradiated CD4^−^ depleted splenocytes. CD4^+^CD25^hi^ T cells from EAE mice (Treg^EAE^), NK treated EAE mice (Treg^NK-EAE^) or IgG-NK treated EAE mice (Treg^IgG-NK-EAE^) were added according to indicated ratios with MOG_35–55_. The intensities of CFSE were determined at day 5 by FACS. CD4^+^CD25^hi^ Treg cells from EAE mice with IgG-NK cell treatment displayed stronger suppressive effect (p< 0.05). Mice: n = 4 per group. (**E**) Treg cells were depleted by injection of 100 µg of anti-CD25 antibody (PC61) intravenously 2 days before EAE induction. 1×10^6^ of IgG-NK cells were injected intravenously at the day of EAE induction. The mean clinical scores of EAE mice with IgG-NK cell treatment were significantly lower but it was reversed by anti-CD25 antibody treatment. Mice: n = 4 per group. Data are representative of at least 2 independent experiments and displayed as the mean ± SEM. *: p<0.05; **: p<0.01, Krusaki-Wallis test.

We next examined the levels of CD4^+^Foxp3^+^ Treg cells in draining lymph nodes of EAE mice after adoptive transfer of IgG-NK cells. We also observed that there is increase of Treg cells after adoptive transfer of IgG-NK cells (Figure 4A, B) but not in the case of adoptive transfer of NK cells not treated with IgG.

### IgG-NK induced Treg cells are suppressive and are responsible for protection from EAE

We investigated the suppressive function of Treg cells in EAE mice after adoptive transfer of IgG-NK cells. We sorted CD4^+^CD25^hi^ Treg cells (>95% express Foxp3, Figure S5) from spleens of EAE mice that had received, or not, an adoptive transfer of untreated NK cells or IgG-NK cells, and cultured these Treg cells with carboxyfluorescein diacetate succinimidyl ester (CFSE) labeled CD4^+^CD25^−^ T cells from spleens of untreated EAE mice, antigen presenting cells (irradiated CD4^−^ splenocytes from naïve mice) and MOG_35–55_. As shown in Figure 4C and D, Treg cells from untreated (Treg^EAE^) and NK cell treated (Treg^NK-EAE^) EAE mice suppressed the cell division of CD4^+^ T cells from 31% to 16.97% and 14.73% respectively (Figure 4D), at a 1∶0.5 ratio of CD4^+^ T cells to Treg cells. While Treg cells from EAE mice with adoptive transfer of IgG-NK cells (Treg^IgG-NK-EAE^) are slightly more potent in suppressing the cell division (11.73±2.36%, p<0.05, Figure 4D).

A previous study reported that the beneficial effect of IgG in EAE depend on increasing Treg cells [15]. We therefore investigated whether IgG-NK cell protection from EAE is dependent on Treg cells. To address this, we depleted Treg cells by using a monoclonal anti-CD25 antibody (clone PC61) in EAE mice with adoptive transfer of IgG-NK cells. More than 90% of Treg cells were depleted in different tissues, i.e. blood, spleen and lymph nodes, as confirmed by flow cytometry (Figure S6). We observed that Treg depletion completely abrogated the protective effect of IgG-NK cells (Figure 4E). Collectively, our data suggest that IgG-NK cells increase the number (Figure 4A and B) and a marginal increase in suppressive function (Figure 4C and D) of Treg cells *in vivo* and that Treg cells are required for the observed protective effect of IgG-NK cells on EAE (Figure 4E).

NK cells also expressed CD25, although the expression level is much lower than Treg cells, so anti-CD25 antibody treatment may also deplete NK cells. To address this issue, we injected mice with anti-CD25 antibody and compared their NK cell percentage to mice that received isotype control at day 3, 5 and 7. We observed that the percentages of NK cells in lymph nodes and spleen were not changed after anti-CD25 antibody treatment (Data not shown).

### IgG-NK cells increase IL-2 production during antigen specific T cell responses and the expression of IL-2 receptor α, CD25, in Treg cells

IL-2 is an important cytokine for maintenance, induction and expansion of CD4^+^CD25^+^ Treg cells [19]. IL-10 and TGF-β1 are also included as they are important in Treg cell induction [20]. To address the mechanism involved in expansion of Treg cells by IgG in EAE mice, we studied the production of IL-2, IL-10 and TGF-β1 during specific T cell responses to MOG_35–55_.

**Figure 5:**
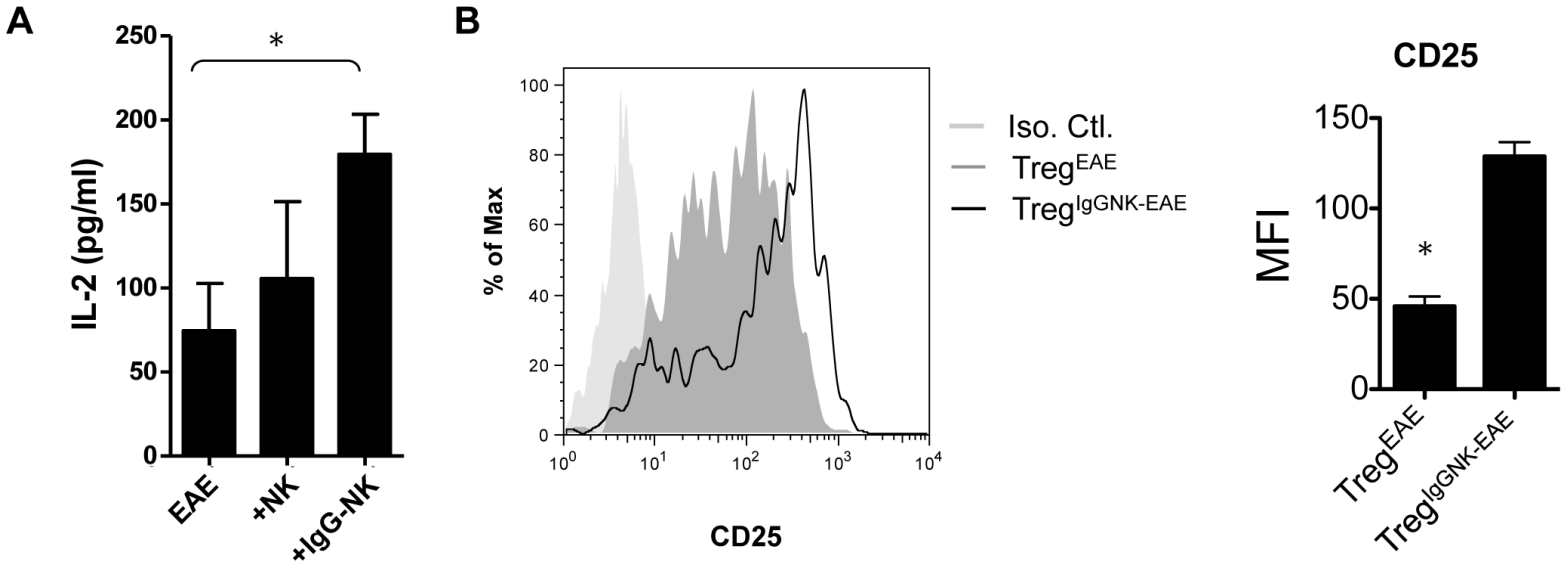
IgG-NK cells induce CD4^+^Foxp3^+^ Treg cells and antigen specific T cells to express higher CD25 and IL-2 respectively. EAE was induced in C57BL/6N mice and 1×10^6^ of IgG-NK cells or untreated-NK cells were injected intravenously at the day of EAE induction. (**A**) 10 days after EAE induction, cells were isolated from draining lymph nodes and 2.5×10^6^ isolated cells were re-stimulated with MOG_35–55_ for 72 hours. Supernatant was collected and the IL-2 levels were studied by ELISA. IL-2 production was significantly increased after adoptive transfer of IgG-NK cells (p<0.05) (n = 5–7). Krusaki-Wallis test. (**B**) Cells were isolated from draining lymph nodes 10 days after EAE induction and studied by FACS. CD4^+^Foxp3^+^ Treg cells from EAE mice (Treg^EAE^) and IgG-NK treated EAE mice (Treg^IgG-NK-EAE^) were gated and the expression of CD25 was shown. Treg^IgG-NK-EAE^ expressed significantly higher CD25 when compared with Treg^EAE^ (n = 4). Data are pooled from 2 independent experiments and displayed as the mean ± SEM. *: p<0.05. Mann-Whitney-U-Test.

After culture with MOG_35–55_, cells from draining lymph nodes of EAE mice that had been infused with IgG-NK cells produced high levels of IL-2 (Figure 5A), while production of TGF-β1 and IL-10 was not appreciably changed (Figure S3). Treg^IgG-NK-EAE^ had higher expression of IL-2 receptor α, i.e. CD25, than Treg^EAE^ (Figure 5B). This indicated that Treg^IgG-NK-EAE^ may be more responsive to IL-2 mediated expansion/survival. All these data suggested that enhanced levels of IL-2 may be involved in the increase of Treg cells observed as a result of IgG-NK cell treatment.

### IgG-NK cells convert naïve T cells to Tregs through a process requiring IL-2 and TGF-β1

**Figure 6:**
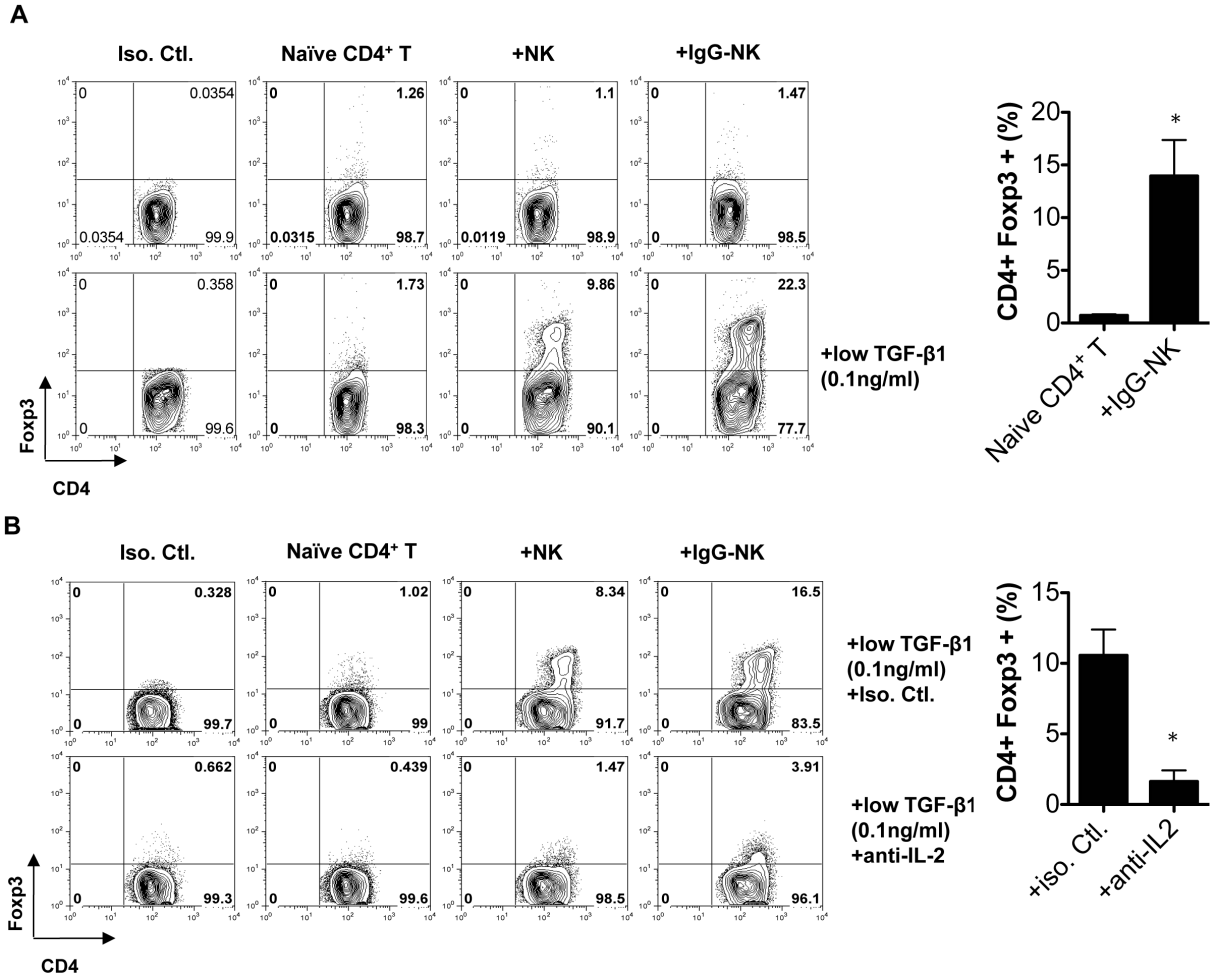
IgG-NK cells induce naïve CD4^+^CD62L^+^ T cells to convert into CD4^+^Foxp3^+^ Treg cells in the presence of IL-2 and TGF-β1. (**A, B**) 1×10^6^ naïve CD4^+^CD62L^+^ T cells purified from spleens of naïve mice were cultured with plate-bound anti-CD3 and soluble anti-CD28 antibodies. 1×10^6^ isolated NK cells or IgG-NK cells were added to the culture with or without low dose of TGF-β (0.1 ng/ml) for 4 days. (**B**) 10 µg/ml of anti-IL-2 antibody or isotype control was added to neutralize IL-2. Data displayed as the mean ± SEM from 4 independent experiments. *: p<0.05. Mann-Whitney-U-Test.

To further investigate the mechanism how IgG-NK cells induce Treg cells, we performed the following *in vitro* experiments. Because TGF-β1 is required for the induction of Treg cells from naïve CD4^+^ T cells in mice [20], we cocultured IgG-NK cells with naïve CD4^+^ T cells and a low dose of TGF-β1 (0.1 ng/ml) with anti-CD3 and anti-CD28 stimulation. The reason for keeping TGF-β1 in low concentration is that high dose of TGF-β1 may mask the need for other factors in induction of Treg cells. After 4 days of culture, naïve CD4^+^ T cells cultured with IgG-NK expressed Foxp3, the lineage-specific transcription factor for Treg cells, in the presence, but not in the absence, of TGF-β1 (Figure 6A). We also observed the increase of Foxp3 expression in CD4^+^ T cells after coculture with IgG-untreated NK cells, although it is not as marked as IgG-NK cells (Figure 6A).

Since there was an increase of IL-2 production in EAE mice with IgG-NK cell treatment and Treg^IgG-NK-EAE^ expressed higher IL-2 receptor α (Figure 5B), we investigated the role of IL-2 in inducing Tregs cells by IgG-NK cells. We blocked IL-2 activity by using IL-2 neutralization antibody. We observed that the Foxp3 expression was significantly reduced (Figure 6B). Therefore, these *in vitro* experiments confirmed that IgG-NK cells induce Treg cells from naïve CD4^+^ T cells in the presence of TGF-β1 and IL-2 and support the conclusion that NK cells are required during IgG treatment (Figure 1) because they are responsible for inducing Treg cells *in vivo* (Figure 3).

### CD4^+^CD25^hi^ Treg cells induced by IgG-NK cells suppressed T cell proliferation

**Figure 7:**
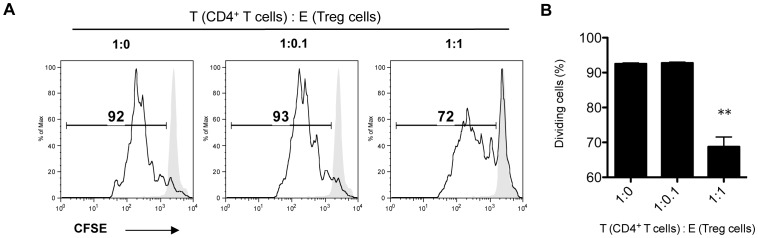
CD4^+^CD25^hi^ Treg cells induced by IgG-NK cells suppresses CD4^+^ T cells proliferation. (**A, B**) CD4^+^CD25^hi^ Treg cells was induced by coculturing IgG-NK cells and CD4^+^CD62L^+^ T cells and sorted by FACS. 2×10^4^ CFSE-labeled CD4^+^CD62L^+^ T cells were cocultured with the sorted CD4^+^CD25^hi^ T cells according to the indicated ratios. Plate bound anti-CD3 and soluble anti-CD28 antibodies were used to stimulate T cell proliferation. The intensities of CFSE were determined at day 4 by flow cytometry. CD4^+^CD25^hi^ Treg cells significantly suppressed CD4^+^ T cells proliferation at 1 to 1 ratio (p<0.01). Representative of 4 independent experiments (**A**); Data pooled from 4 independent experiments (**B**).

To investigate the inhibitory function of the Treg cells induced by IgG-NK cells, sorted CD4^+^CD25^hi^ Treg cells were cocultured with CFSE labeled naïve CD4^+^CD62L^+^ T cells. Plate bound anti-CD3 and anti-CD28 antibodies were used to stimulate naïve T cells proliferation. We observed that IgG-NK cell induced Treg cells significantly suppressed naïve T cell proliferation (Figure 7).

## Discussion

In this study, we report 2 novel findings. First, we demonstrated that the protective effect of IgG in the development of EAE in mice depends on the presence of NK cells. This observation may be of relevance to treatment of human autoimmune diseases by IVIG. The percentage of NK cells in circulating lymphocytes varies from ∼5 to ∼20% in humans [21] and the level of NK cells could conceivably constitute a contributing factor for the efficacy of IVIG treatment.

The induction of CD4^+^Foxp3^+^ Treg cells by IgG-NK cells is our second novel finding. By *in vitro* study, we presented evidence that IgG-NK cells are able to induce CD4^+^Foxp3^+^ Treg cells from naïve CD4^+^ T cells and that it is dependent on IL-2 and TGF-β1 (Figure 6). Although we identified the cytokines required for inducing Treg by NK cells, T cell co-stimulatory molecules expressed by NK cells may also be involved in the induction. IL-2 and plate-bound anti-CD16 activate NK cells to express CD86 for co-stimulating IL-2 production and proliferation in CD4^+^ T cells [9], [22]. Plate-bound anti-CD16 activates NK cells to express another T cell co-stimulatory molecule, i.e. OX40-ligand [22], which can deliver a survival signal to Treg cells [23]. However, IgG did not induce NK cells to express CD86 and OX40-ligand (data not shown), excluding the role of these two co-stimulatory molecules in inducing Treg by IgG-NK cells. Interestingly, IL-2-activated NK cells are shown to suppress Foxp3 expression in CD4^+^CD25^−^ T cells, which is different from our observation that IgG-NK cells induce Foxp3 expression [24]. These suggest that upon different stimulations, NK cells display distinct regulatory roles in Treg induction.

An alternative mechanism underlying the role of NK cells in the efficacy of IVIG treatment was recently proposed on the basis of *in vitro* studies [25]. Tha-In et al. demonstrated that NK cells suppress T-cell priming by mediating antibody-dependent cellular cytotoxicity (ADCC) to IVIG-treated DCs and this ADCC is induced by IgG via CD16, the low affinity Fcγ receptor, on NK cells [25]. Although we have demonstrated that NK cells induce Treg cells to ameliorate EAE during IgG treatment (Figure 1 and 3), our data in no way exclude direct killing of IgG-primed antigen presenting cells (APCs) by NK cells as another mechanism through which NK cells could suppress autoimmune disease, since this could inhibit the presentation of autoantigen to self-reactive T cells. Future *in vivo* studies are needed to examine the possible role of ADCC-driven elimination of APCs by NK cells in IgG treatment.

IVIG can also directly regulate NK cells cytotoxicity and cytokine production. Recent studies demonstrated that IVIG suppresses NK cells cytotoxicity towards two cancer cell lines, i.e. K562 and A431 by exhausting the NK cell cytotoxicity machinery via spontaneous degranulation. IVIG also activates them to produce more IFN-γ [26], [27]. This may contribute to the therapeutic effect of IVIG because IFN-γ suppresses Th17 responses, which is critical in promoting autoimmune diseases [28]. These studies also demonstrated that the number of peripheral NK cells number drop significantly in patients after IVIG treatment [26], [27], indicates that IVIG may induce the tissue redistribution of NK cells, as they express different chemokine receptors for migration to inflammatory site and secondary lymphoid organs [29].

A number of studies that investigated the function of NK cells in EAE reported contradictory results [30]–[33]. Hao et al. [30] recently reported that NK cells are required to suppress Th17 induction by microglia in CNS of EAE mice. However, Winkler-Pickett et al. [33] demonstrated that depletion of NK cells is protective in EAE mice and is associated with less DCs maturation and a reduction in pathogenic T cells accumulation. In the present study we did not observe a discernible difference in clinical scores between the EAE mice with or without NK cell depletion (Figure 1A). This can be explained by the timing of NK cell depletion in these studies [30]–[33]. As we only focus on the period during IgG treatment (2 doses on day 0 and 4), we only depleted NK cells by anti-asialos GM-1 antibody 1 day before the administration of IgG (2 doses on day −1 and 3), limiting the period of NK deficiency to coincide with IgG treatment. The other studies depleted NK cells throughout the entire experiments, affecting also the later period where NK cells could be involved in effector mechanisms of disease during later phases of EAE development.

Direct stimulatory effects of Treg cells by IVIG *in vitro* were also reported [15], [34]. Ephrem et al. (2008) showed that mouse Treg cells increase their proliferation when cultured with IVIG. Another study also reported that coculture of human Treg cells with IVIG enhance their TGF-β and IL-10 production [34]. We cannot exclude the possibility that IVIG could directly stimulate Treg cells *in vivo*, however, we demonstrated that if NK cells were depleted, the beneficial effect of IgG on disease as well as the expansion of Treg cells *in vivo* are prevented (Figures 1 and 3). These results lead to the conclusion that NK cells mediate the effect of IVIG treatment in suppressing EAE at least in part by inducing Treg cells ***in vivo***.

We observed that NK cells play a critical role in ameliorating EAE by IgG treatment, with lower Th1 and Th17 immune response (Figure 2B) and increased Treg cells (Figure 4A and B) in the draining lymph nodes. However, CNS is unique compared to peripheral compartment [35] and how NK cells regulate T cell response in CNS during IgG treatment on EAE mice should be investigated in the future.

Interestingly, we observed that NK cell depleted mice produced more IL-17 after treatment with IgG (Figure 1B). Innate IFN-γ has been implicated in suppressing Th17 response and related tissue-specific autoimmunity. Depletion of NK cells, which are the major source of innate IFN-γ, may lead to upregulation of IL-17 response in IgG-treated mice. However, similar phenomenon was not observed in NK cell depleted mice without IgG treatment (Figure 1B). Further investigation is required to study the effect of IgG in IL-17 response in NK cell depleted mice.

We demonstrated that Treg cells are required for the observed protective effect of IgG-NK cells on EAE by depleting Treg cells in IgG-NK treated group with anti-CD25 antibody (Figure 4E). Previous study also demonstrated that Treg cells are critical to suppress EAE by IVIG [15]. Although the use of anti-CD25 antibody to deplete Treg cells is widely accepted, it should be noted that most of the activated lymphocytes, including effector T cells, activated NKT cells and NK cells expressed CD25. Ligation of CD25 may result in cellular depletion and/or functional modulation on these cells.

IVIG has been shown to be effective against different autoimmune diseases. Although we and others clearly demonstrated that IgG and IVIG suppresses EAE [2], [25], therapeutic efficacy of IVIG in treating multiple sclerosis is still controversial. EAE is used for the experimental model for multiple sclerosis because they have similar pathological features, including CNS inflammation and demyelination. However, EAE is an artificial animal model with different immunopathogenic mechanisms to multiple sclerosis. It is known that EAE is mainly CD4^+^ Th1 and/or Th17 pathogenic T cells driven, but CD8^+^ T cells are much more dominant in multiple sclerosis [36]. Nevertheless, we demonstrated that IgG ameliorates EAE and its efficacy is dependent on NK cells. Future studies are required to translate this observation into multiple sclerosis and other human autoimmune diseases.

We demonstrated that the increased induction of Foxp3^+^ Treg cells from naïve T cells by IgG-NK cells is depended on IL-2 by using IL-2 neutralization antibody (Figure 6). Treg cells constitutively express high level of high affinity IL-2 receptor, CD25, while NK cells usually expresse low affinity IL-2 receptor [37]. High level of IL-2 is crucial for Treg growth, survival and expansion [38]. IL-2 also induces NK cells activation and proliferation [39]. Therefore, neutralizing IL-2 in NK-T cells coculture may affect both NK and T cells and resulted in lower Treg conversion.

IVIG consists of the polyclonal IgG pooled from the sera of different donors, including different polyreactive natural antibodies and antigen specific antibodies. During the production process, IgG are modified and stabilizers are also used to prevent IgG aggregation that may trigger non-specific complement activation or other pro-inflammatory response. In this study, we used unmodified human IgG in our EAE model. Although there are possibilities that our IgG is more “proinflammatory” when compared to IVIG, we demonstrated that it suppressed EAE development (Figure S7) and induced Foxp3^+^ Treg cells in EAE mice (Figure 3), similar to IVIG [15].

In conclusion, we report a pivotal role for NK cells in suppressing EAE by IgG treatment through induction of Treg cells. Future studies should be carried out to confirm our observation in humans being treated for autoimmune diseases by IVIG and this may provide further hints in how best to use IVIG as a therapeutic. Importantly, we identify a novel function of NK cells in peripheral tolerance as inducers of CD4^+^Foxp3^+^ Treg cells. These observations provide a new insight into the role of NK cells in regulating adaptive immunity, immune tolerance and hence autoimmune diseases.

## Materials and Methods

### EAE induction and assessment

Eight to twelve weeks old C57BL/6N mice obtained from Laboratory of Animal Unit, The University of Hong Kong were used in the experiments. In some experiments, C57BL/6 mice were purchased from The Jackson Laboratory (Bar Harbor, ME). These mice were kept in a specific pathogen-free facility in National Institues of Health. EAE was induced by subcutaneous injection of 200 µl of emulsion containing 200 µg of myelin oligodendrocyte glycoprotein peptide fragment 35–55 (MOG_35–55_) (MEVGWYRSPFSRVVHL-YRNGK) (Chinese Peptide Company, Zhejiang, China) in complete Freund's adjuvant with 400 µg of H37Ra Mycobacterium tuberculosis (Difco Laboratories, L'Arbresk, France). 400 ng of pertussis toxin (Calbiochem, San Diego, CA) was injected intravenously on day 0 and 2. All procedures were approved by the Committee on the Use of Live Animals in Teaching and Research of the University of Hong Kong and by the Animal Care and Use Committee of the National Eye Institute.

Disease severity was assessed daily in a blinded fashion as described previously [40]. Briefly, scores were assigned as follows: 0: no clinical sign; 1: weakness of the tail; 2: complete tail paralysis; 3: partial hind limb paralysis; 4: complete hind limb paralysis; 5: incontinence and partial or complete paralysis of forelimbs; 6: death.

### IgG treatment

10% IgG was freshly prepared from human serum IgG (Calbiochem, San Diego, CA) in PBS. This IgG is bioequivalent to the commercially available IVIG (Gamunex, Talecris Biotherapeutics) in terms of suppressing EAE (Figure S7). Human IgG or commercially available IVIG was injected intravenously on the same day and 4 days after immunization at a dose of 1g/Kg [14].

### In vivo depletion of NK cells and Treg cells

To deplete NK cells *in vivo*, mice were injected intravenously with 50 µl of anti-asialo GM1 antibody (Wako, Richmond, VA) 1 day before and 3 days after immunization. For Treg depletion, 100 µg of anti-CD25 antibody, PC61 (Biolegend, San Diego, CA), in 100 µl of PBS was injected intravenously 2 days before immunization.

### Adoptive transfer of NK cells

NK cells were negatively selected from splenocytes by NK Isolation Kit (Miltenyi Biotec, Bergisch Gladbach, Germany). They were cultured with or without 10 mg/ml of IgG (Calbiochem) for 18 hours and then the NK cells were washed with PBS for 2 times to remove the IgG. 1×10^6^ of untreated NK cells or IgG-NK cells were injected intravenously on the same day of immunization.

### Measurement of secreted cytokines

Cells from draining lymph nodes of the site of immunization were isolated 10 days after EAE induction. 2.5×10^6^ cells were cultured with 20 µg/ml of MOG_35–55_ in 1 ml of RPMI containing 10% of fetal calf serum (FCS) for 72 hours. Supernatants were collected and the levels of cytokines were determined by Flowcytomix (Bender Medsystems, Vienna, Austria).

### Surface and intracellular staining of Treg cells for flow cytometry analysis

Cells were surface stained with anti-mouse CD4 FITC (RM4-5) and anti-mouse CD25 APC (PC61.5), fixed and permeabilized according to the manufacturer's protocol (Anti mouse Foxp3 staining set, eBioscience, San Diego). Then, cells were stained for intracellular Foxp3 PE (150D/E4). All antibodies were purchased from eBioscience. Treg cells were identified by Foxp3 expression in gated CD4 cells.

### T cell suppression assay

CD4^+^CD25^−^ T cells, CD4^+^CD62L^+^ T cells and CD4^+^CD25^hi^ T cells were sorted by FACSAria. CD4^+^CD25^−^ T cells and CD4^+^CD62L^+^ T cells were labeled with CFSE before activation. 2×10^4^ labeled CD4^+^CD25^−^ T cells were activated with 1×10^4^ irradiated CD4^−^depleted splenocytes with MOG_35–55_. 2×10^4^ labeled CD4^+^CD62L^+^ T cells were activated with plate bound anti-CD3 and anti-CD28 antibodies. CD4^+^CD25^hi^ T cells were added at the indicated ratios for 4–5 days. Cell division by CFSE dilution was analyzed.

### *In vitro* induction of CD4^+^Foxp3^+^ cells

Naïve CD4^+^CD62L^+^ T cells were isolated from the spleen of naïve C57BL/6N mice by CD4^+^CD62L^+^ T Cell Isolation Kit II (Miltenyi). 1×10^6^ isolated naïve CD4^+^CD62L^+^ T cells were activated with plate-bound anti-CD3 and soluble anti-CD28 antibodies. 1×10^6^ isolated untreated NK cells or IgG-NK cells were added to the culture with or without TGF-β (0.1 ng/ml) for 4 days. In some cases, 10 µg/ml of anti-IL-2 antibody or isotype control antibody was added to the culture.

### Perfusion and Tissue Processing

At the end of the experiment (day 15), the animals were given a lethal dose of sodium pentobarbital and perfused intracardially with normal saline followed by perfusion with 200 to 300 ml of fixative containing 4% paraformaldehyde in 0.1 M phosphate buffer (pH 7.4). The lumbar spinal cords were harvested and post-fixed in 2% paraformaldehyde with 2.5% glutaraldehyde overnight at 4°C. In the following day, samples were treated with 1% osmium tetroxide for 4 hours, dehydrated in a series of graded ethanol, cleared in propylene oxide and embedded in pure Epon. One micrometer semi-thin sections were cut by an ultramicrotome (Leica, Germany), mounted on gelatin-coated glass slides and stained with 1% toluidine blue to visualize the myelin.

### Statistical analysis

Mann-Whitney-U-Test was used for two groups comparison and Krusaki-Wallis test was used when more than two groups are shown. P value of 0.05 was used to indicate significance.

## Supporting Information

Figure S1 **Depletion of NK cells in vivo by anti-asialo GM1 antibody.** NK cells were depleted as described in Methods and Materials. The percentage of NK cells in different tissues, i.e. blood, lymph node and spleen, were determined at different time point. Nearly 90% of NK1.1+CD3− NK cells were depleted. Data are displayed as mean ± SEM of 4 mice per each time point.
(TIF)

Figure S2 **Comparison of IgG and BSA in treating EAE.** EAE was induced as described in Materials and Methods. IgG and BSA was injected intravenously at day 0 and 4 (n = 10). Data are pooled from 2 independent experiments.
(TIF)

Figure S3 **Cytokines productions of EAE mice.** EAE was induced in C57BL/6 mice. Untouched NK or IgG-NK cells were adoptive transferred as described in Methods and Materials. At day 10, cells were isolated from draining LNs. 2.5×106 isolated cells were re-stimulated with MOG35–55 (20 µg/ml) for 72 hours. Cytokine productions were determined. Data are displayed as mean ± SEM from 2 independent experiments with total of 4–7 mice per group.
(TIF)

Figure S4 **Demyelination is reduced in EAE mice after IgG or IgG-NK cell treatment.** Toluidine blue staining of 1 µm semi-thin sections were used to visualize myelination of EAE mice with different treatments at day 15: (A) Untreated EAE mice; EAE mice treated with (B) IgG; (C) anti-asialo GM1 antibody; (D) IVIG and anti-asialo GM1 antibody; (E) untouched NK cells; (F) IgG-NK cells. When compared with (A) untreated EAE mice, mice treated with (B) IgG or (F) IgG-NK cell showed decrease in demyelination. The scale bar is 400 µm for A–F, 80 µm for A1–F1, 15 µm for A2–F2. Representative of 4 mice for each condition.
(TIF)

Figure S5 **Expression of Foxp3 in sorted CD4+CD25hi T cells.** EAE was induced in naïve mice. Untouched NK or IgG-NK cells were adoptive transferred as described in Methods and Materials. At day 10, cells were isolated from spleen. CD4+CD25hi T cells were sorted and stained for Foxp3. More than 95% of these sorted cells were Foxp3^+^.
(TIF)

Figure S6 **Depletion of Treg cells in vivo by anti-CD25 antibody (PC61).** Treg cells were depleted as described in Methods and Materials. The percentage of CD4^+^Foxp3^+^ cells in different tissues, i.e. blood, lymph node and spleen, were determined at different time point. Nearly 90% of CD4^+^Foxp3^+^ Treg cells were depleted. Data are displayed as mean ± SEM of 4 mice per each time point.
(TIF)

Figure S7 **Comparison of IVIG from different sources in treating EAE.** IVIG prepared from Calibochem is bioequivalent to commercially available IVIG, Gamunex, in suppressing EAE development (n = 10). Data are pooled from 2 independent experiments.
(TIF)
