# Supplementary figures and images for: Essential Role of NK Cells in IgG Therapy for Experimental Autoimmune Encephalomyelitis
Source: PLoS One. 2013 Apr 5;8(4):e60862. doi: 10.1371/journal.pone.0060862 (PMC3618232; doi:10.1371/journal.pone.0060862)

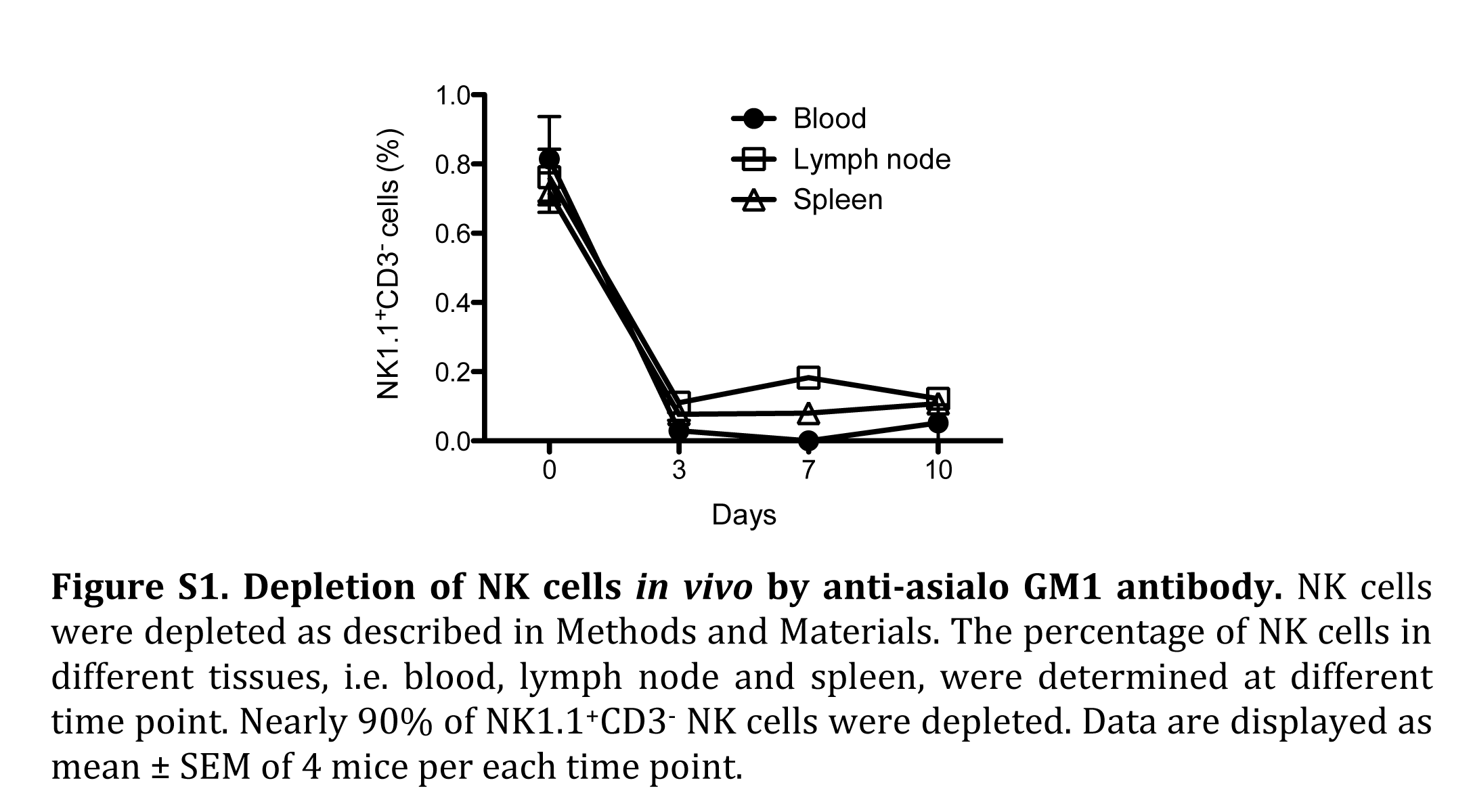

Supplement: Figure S1 — Depletion of NK cells in vivo by anti-asialo GM1 antibody. NK cells were depleted as described in Methods and Materials. The percentage of NK cells in different tissues, i.e. blood, lymph node and spleen, were determined at different time point. Nearly 90% of NK1.1+CD3− NK cells were depleted. Data are displayed as mean ± SEM of 4 mice per each time point. (TIF) [file pone.0060862.s001.tif]

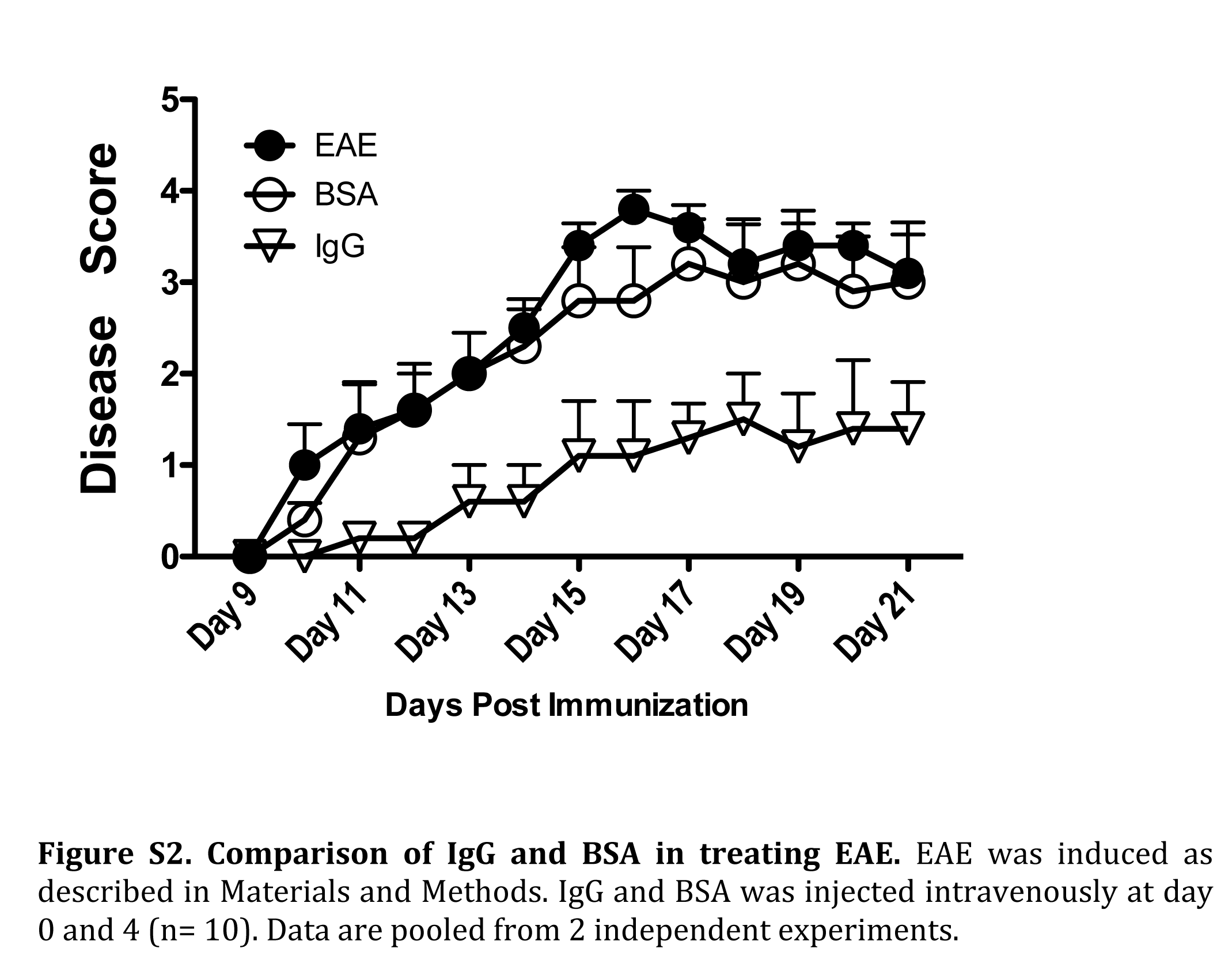

Supplement: Figure S2 — Comparison of IgG and BSA in treating EAE. EAE was induced as described in Materials and Methods. IgG and BSA was injected intravenously at day 0 and 4 (n = 10). Data are pooled from 2 independent experiments. (TIF) [file pone.0060862.s002.tif]

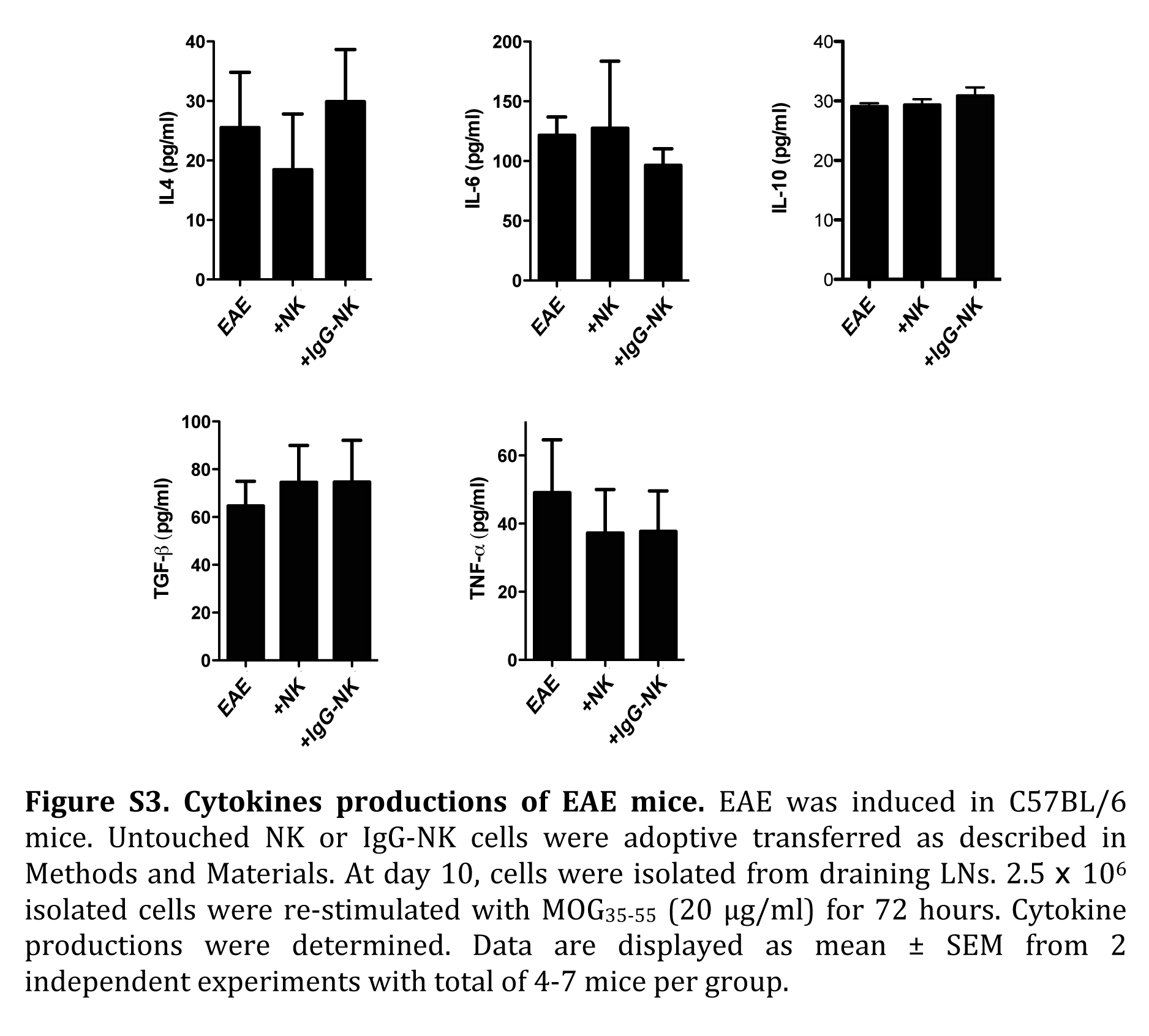

Supplement: Figure S3 — Cytokines productions of EAE mice. EAE was induced in C57BL/6 mice. Untouched NK or IgG-NK cells were adoptive transferred as described in Methods and Materials. At day 10, cells were isolated from draining LNs. 2.5×10^6^ isolated cells were re-stimulated with MOG35–55 (20 µg/ml) for 72 hours. Cytokine productions were determined. Data are displayed as mean ± SEM from 2 independent experiments with total of 4–7 mice per group. (TIF) [file pone.0060862.s003.tif]

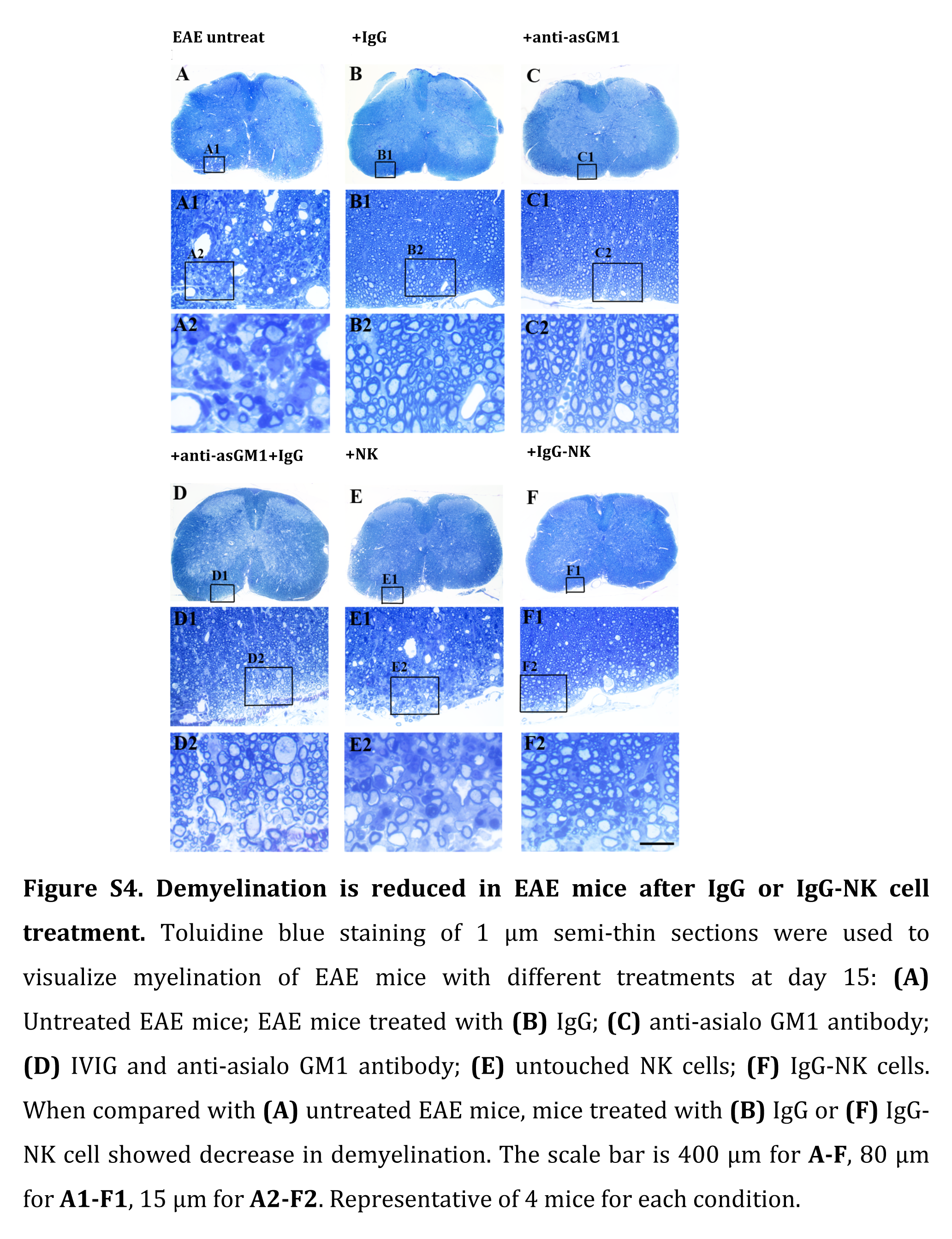

Supplement: Figure S4 — Demyelination is reduced in EAE mice after IgG or IgG-NK cell treatment. Toluidine blue staining of 1 µm semi-thin sections were used to visualize myelination of EAE mice with different treatments at day 15: (A) Untreated EAE mice; EAE mice treated with (B) IgG; (C) anti-asialo GM1 antibody; (D) IVIG and anti-asialo GM1 antibody; (E) untouched NK cells; (F) IgG-NK cells. When compared with (A) untreated EAE mice, mice treated with (B) IgG or (F) IgG-NK cell showed decrease in demyelination. The scale bar is 400 µm for A–F, 80 µm for A1–F1, 15 µm for A2–F2. Representative of 4 mice for each condition. (TIF) [file pone.0060862.s004.tif]

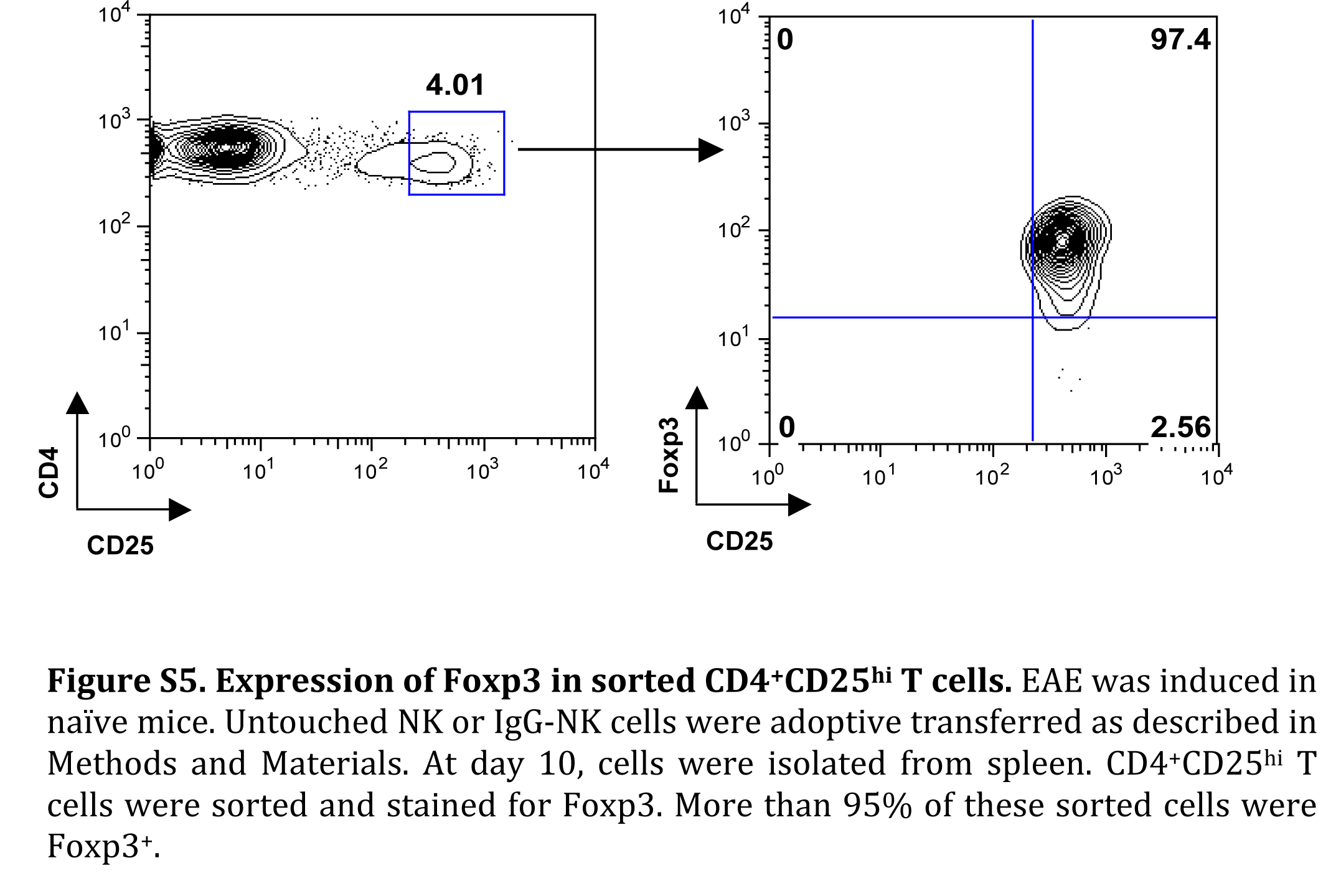

Supplement: Figure S5 — Expression of Foxp3 in sorted CD4+CD25hi T cells. EAE was induced in naïve mice. Untouched NK or IgG-NK cells were adoptive transferred as described in Methods and Materials. At day 10, cells were isolated from spleen. CD4+CD25hi T cells were sorted and stained for Foxp3. More than 95% of these sorted cells were Foxp3+. (TIF) [file pone.0060862.s005.tif]

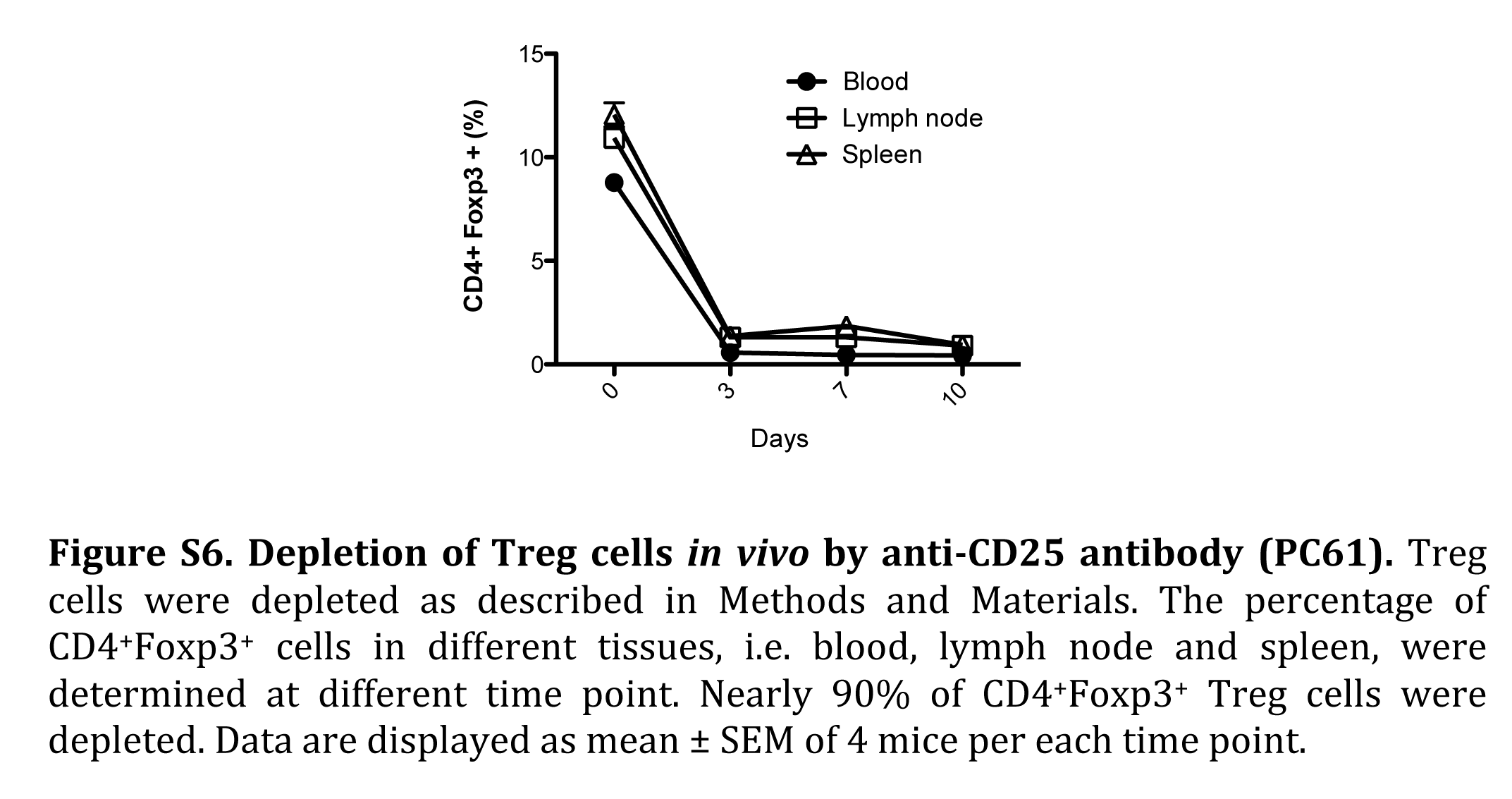

Supplement: Figure S6 — Depletion of Treg cells in vivo by anti-CD25 antibody (PC61). Treg cells were depleted as described in Methods and Materials. The percentage of CD4+Foxp3+ cells in different tissues, i.e. blood, lymph node and spleen, were determined at different time point. Nearly 90% of CD4+Foxp3+ Treg cells were depleted. Data are displayed as mean ± SEM of 4 mice per each time point. (TIF) [file pone.0060862.s006.tif]

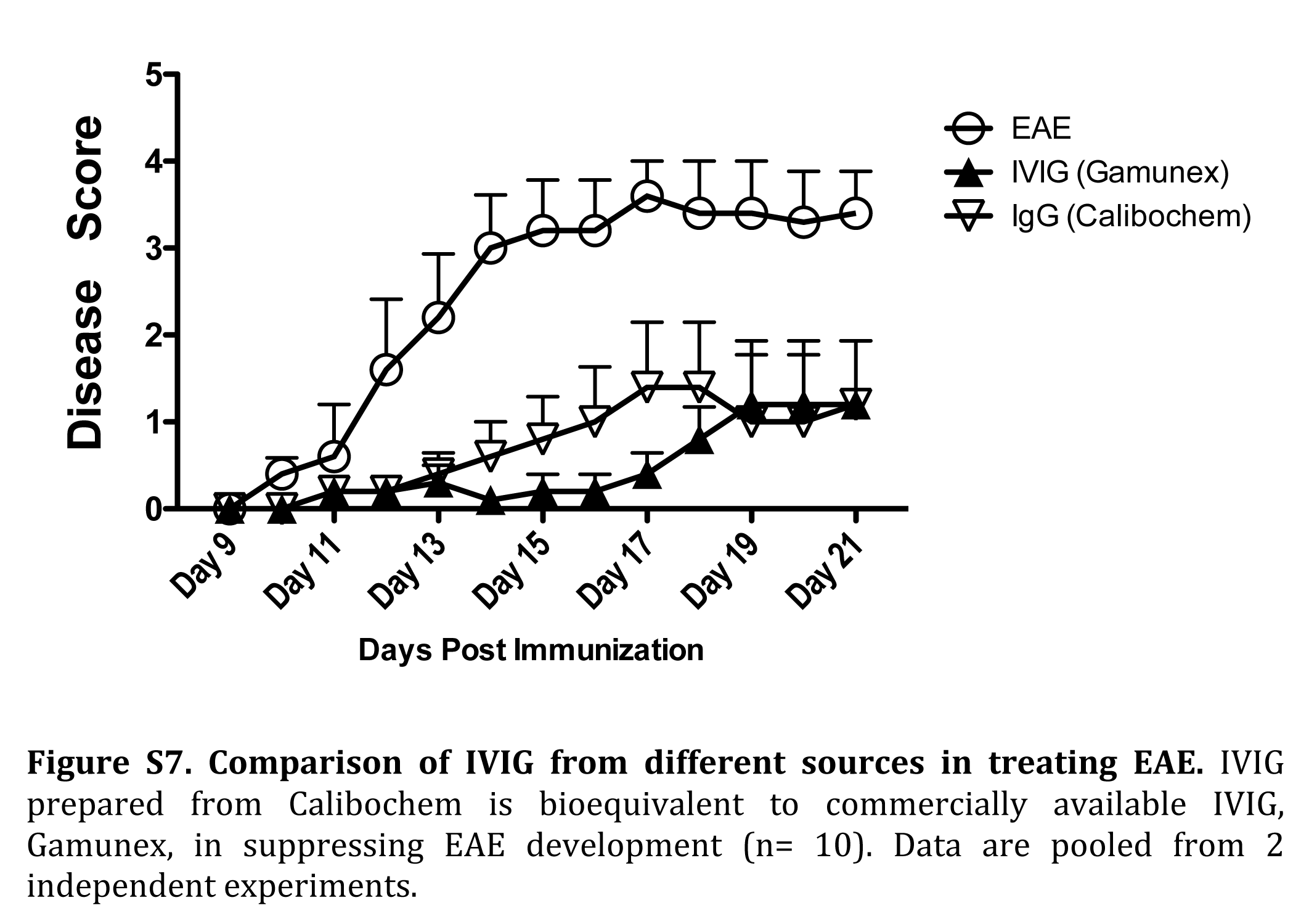

Supplement: Figure S7 — Comparison of IVIG from different sources in treating EAE. IVIG prepared from Calibochem is bioequivalent to commercially available IVIG, Gamunex, in suppressing EAE development (n = 10). Data are pooled from 2 independent experiments. (TIF) [file pone.0060862.s007.tif]
